# System of Implantable Electrodes for Neural Signal Acquisition and Stimulation for Wirelessly Connected Forearm Prosthesis

**DOI:** 10.3390/bios14010031

**Published:** 2024-01-09

**Authors:** Octavian Narcis Ionescu, Eduard Franti, Vlad Carbunaru, Carmen Moldovan, Silviu Dinulescu, Marian Ion, David Catalin Dragomir, Carmen Marinela Mihailescu, Ioan Lascar, Ana Maria Oproiu, Tiberiu Paul Neagu, Ruxandra Costea, Monica Dascalu, Mihai Daniel Teleanu, Gabriela Ionescu, Raluca Teleanu

**Affiliations:** 1 Faculty of Mechanical and Electrical Engineering, Petroleum and Gas University from Ploiesti, 100680 Ploiesti, Romania; octavian.ionescu@imt.ro (O.N.I.); ionescu_g_r@yahoo.com (G.I.); 2 National Institute for Research and Development for Microtechnology Bucharest, 077190 Bucharest, Romania; carmen.moldovan@imt.ro (C.M.); silviu.dinulescu@imt.ro (S.D.); marian.ion@imt.ro (M.I.); david.dragomir@imt.ro (D.C.D.); carmen.mihailescu@imt.ro (C.M.M.); 3 ICIA, Centre of New Electronic Architectures, 061071 Bucharest, Romania; monica.dascalu@upb.ro; 4 Emergency Clinic Hospital Bucharest, 014461 Bucharest, Romania; carbunaru.vlad@gmail.com (V.C.); ioan.lascar@umfcd.ro (I.L.); anamaria.oproiu@umfcd.ro (A.M.O.); paul.neagu@umfcd.ro (T.P.N.); 5 University of Medicine and Pharmacy UMF Carol Davila, 050474 Bucharest, Romania; daniel.teleanu@umfcd.ro (M.D.T.); raluca.teleanu@umfcd.ro (R.T.); 6 Faculty of Veterinary Medicine, University of Agricultural Sciences and Veterinary Medicine of Bucharest, 011464 Bucharest, Romania; ruxandra.costea@fmvb.usamv.ro; 7 Faculty of Electronics, Telecommunications and Information Technology, National University of Science and Technology Politehnica of Bucharest, 060042 Bucharest, Romania

**Keywords:** plug electrodes, selective acquisition, nerve pulses, forearm prosthesis, neural control

## Abstract

There is great interest in the development of prosthetic limbs capable of complex activities that are wirelessly connected to the patient’s neural system. Although some progress has been achieved in this area, one of the main problems encountered is the selective acquisition of nerve impulses and the closing of the automation loop through the selective stimulation of the sensitive branches of the patient. Large-scale research and development have achieved so-called “cuff electrodes”; however, they present a big disadvantage: they are not selective. In this article, we present the progress made in the development of an implantable system of plug neural microelectrodes that relate to the biological nerve tissue and can be used for the selective acquisition of neuronal signals and for the stimulation of specific nerve fascicles. The developed plug electrodes are also advantageous due to their small thickness, as they do not trigger nerve inflammation. In addition, the results of the conducted tests on a sous scrofa subject are presented.

## 1. Introduction

**Figure 1:**
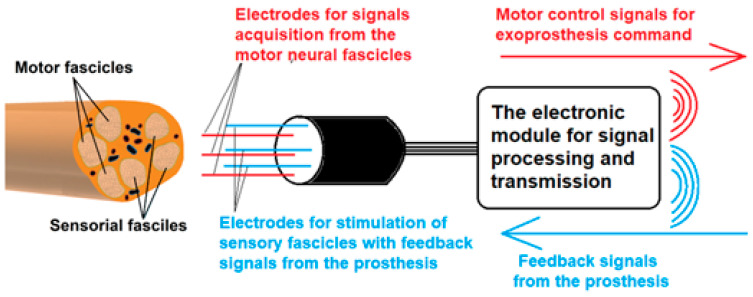
Conceptual design of the developed system, which provides a bidirectional connection with the wirelessly connected prosthesis.

In this article, we present our work regarding the development of a new concept for a complex system consisting of specially designed plug electrodes, frontend electronics, a communication system, and a wireless power supply able to provide both the selective acquisition of neuronal signals and the stimulation of specific nerve fascicles. The conceptual design of the system is presented in Figure 1.

The novelty of our proposal and the advantages that are brought to the prosthetic market are underlined by the state-of-the-art analysis presented herein. Progress in micro- and nanotechnology has generated a large range of active implantable medical devices: implantable brain stimulators, neurostimulators, neural implants for limb prosthesis, insulin pumps, implantable cardioverter defibrillators (transvenous and subcutaneous), cardiac pacemakers, ventricular assist devices, implantable hearing devices, etc. In the last decade, active implantable medical devices have begun to play an important role in treating and even curing many diseases that would otherwise be incurable. As expected, the global market for implantable electronic devices has grown exponentially in recent years: approximately USD 18.42 billion in 2017 and an expected increase to USD 26.75 billion in 2025 [1]. The United States controls about 40% of the global market, followed by Europe (25%), Japan (15%), and the rest of the world (20%). The largest market shares in Europe belong to Germany, Italy, and France [1].

One of the active implantable medical devices that have aroused the increasing interest of researchers in the medical and nanotechnology fields is neural implants. Neural implants are generally used to stimulate specific brain areas or specific nervous pathways for the treatment of diseases such as Parkinson’s, epilepsy, peripheral neuropathy, etc. Neural implants contain microelectrodes, an electronic signal processing module, a transmission/reception module, and a power supply module. The performance of neural implants depends not only on the high selectivity of the electrodes but also on the performance of the data transmission/reception module and on the autonomy provided by the power supply module.

An important field in which neural implants can ensure significant progress is prostheses with neural control for limb amputees. Neural-controlled prostheses usually employ signals acquired from the nerves in the amputation stump to carry out movements. Some neural prostheses have pressure sensors mounted on the fingers, and the signals from these are sent to the neural implants to stimulate the sensory fascicles and generate tactile sensations for the users when handling objects of different shapes and sizes with the prosthesis. Neural implants started being used in 2001 for commanding limb prostheses with neuronal signals [2] and in 2005 for generating tactile sensations in amputees [3,4], but until now, there have been no prostheses where the amputee can move each finger separately and have tactile sensations in each finger. Neural implants for prostheses contain microelectrodes, an electronic signal processing module, a transmission/reception module, and a power supply module. They have different structures depending on the solutions implemented to solve four major restrictions:

1. Microelectrodes for neuro-signal acquisition or for nerve stimulation;
2. Power supply;
3. Data transmission;
4. Biosafety/electrical safety.

The implantable neural microelectrodes are strongly connected with the biological tissue and they play an important role in the overall efficiency of acquisition of the neuronal signals and for the stimulation of specific nerve fascicles. They must have the following characteristics: lightweight, good selectivity, small dimensions that reduce tissue damage, strong conformity with neural tissues, biocompatible, mechanical/electrical safety, and low fabrication costs. Based on these requirements, various electrodes have been developed to perform electrical stimulation and electrophysiological signal recording for prosthesis commands. Among these electrodes, neural probes made by the University of Michigan and electrode arrays made by the University of Utah were widely used in central nerve prostheses applications [5,6], while longitudinal intrafascicular (LIFEs) electrodes were commonly applied in peripheral nerve and intramuscular studies [7]. Starting in 2012, dense electrode arrays and tenuous electrodes were developed to perform complex and precise electro-physiological studies, as they can provide excellent spatial selectivity and have low power consumption [8].

For connecting the peripheral nervous system with prostheses, four categories of microelectrodes are generally used: regenerative electrodes, intra-fascicular electrodes, inter-fascicular electrodes, and extra-neural electrodes [9,10,11].

- Regenerative peripheral neural electrodes are designed to interface peripheral nerve fibers that have regenerated through or past their geometry [12];
- Interfascicular electrodes are designed to gain increased access to the neurons without penetrating the perineurium around the fascicles [13];
- Intrafascicular electrodes provide access to small groups of axons within a peripheral nerve fascicle [7];
- Extraneural electrodes are wrapped around nerves and they are the least invasive electrodes [14]. There are four major types of extraneural electrodes: the epineural electrode, the helical electrode, the book electrode, and the cuff electrode [15,16].

The electrodes used for the peripheral nervous system must have the following properties: flexibility, excellent electrical properties, high spatiotemporal precision, biocompatibility, long-term stability, thin dimensions, and safety for the nerve and surrounding tissues [9,10].

The electrodes, together with the electronic module, transform the neural signals acquired from the nerve into technical signals that will be transmitted to an external device (exoprosthesis, exoskeleton, etc.). The size of the electrodes should be as small as possible, but not smaller than 4 microns so that the impedance is maintained between 0.1 and 2 MΩ [17,18,19]. The impedance varies inversely proportional to the diameter of the electrodes.

**Figure 2:**
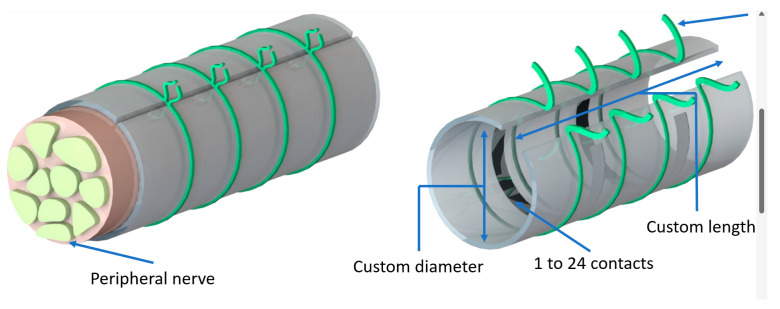
General view of the cuff electrodes.

Some of the most used neural microelectrodes for both recording and stimulation are cuff microelectrodes which are presented in Figure 2. They are extraneural electrodes that can provide a simultaneous interface with many axons in the nerve. This type of electrode was patented and fabricated for the first time in 1984 by Naples et al. [20]. Since then, they have been used in implantable interfaces for many types of medical applications [21]:

- Stimulation and recording the neural activity in the peripheral nervous system [22];
- Acquisition of neural signals for the control of motor exoprostheses [23,24];
- Stimulation of the optic nerve for visual prosthesis [25];
- Electrical stimulation of the nerves, for the restoration of motor functions [26,27].

There is a major inconvenience related to the cuff microelectrodes: this type of electrodes cannot acquire signals from a certain motor fascicle of the nerve, and, consequently, cannot provide the required selectivity for a performant arm prosthesis. A more suitable solution, which could provide the selectivity could be the intra-fascicular electrodes; however, this type of electrode is more difficult to produce and more challenging to implant since each needle should target a specific nerve fascicle.

Because they are used in implantable devices, neural electrodes must be fabricated exclusively with biocompatible materials. The implantable electrodes must have appropriate mechanical, physical, and chemical properties to attenuate the body’s reaction to foreign bodies. They should maintain their properties in the conditions of all the chemical reactions that occur after implantation at the tissue–electrodes interface [28,29]. Different authors recommend polymers for the substrate material and gold, platinum, tungsten, or stainless steel for the electrodes [30,31,32,33,34]. Other options for the electrodes, reported in recent articles, are iridium, tantalum pentoxide, and titanium nitride [35,36].

The implantable electrodes are exposed to very corrosive agents, arising from the chemical reactions within the human body (the high level of oxygen, macromolecules, and saline electrolytes from the tissues where the electrodes are implanted). Typical destructive effects are the detaching of the electrodes from the substrate or the corrosion of the metal pathways [36].

There is no general rule for the production of cuff electrodes, as they have to be adapted specifically to the type of tissue where implanted. The compatibility of the implant with the physicochemical characteristics of the tissues reduces the osteoblasts’ production. Otherwise, the tissues will massively produce osteoblasts that will cover all parts of the electrodes and affect their proper functioning [36,37]. In addition to the properties of the cuff electrodes, it is also important to implant them in such a way as to avoid damage to the nerves or tissues and thus reduce the risk of neuroinflammatory reactions [36,37,38].

## 2. Materials and Methods

### 2.1. Plug Electrodes: Design and Fabrication

**Figure 3:**
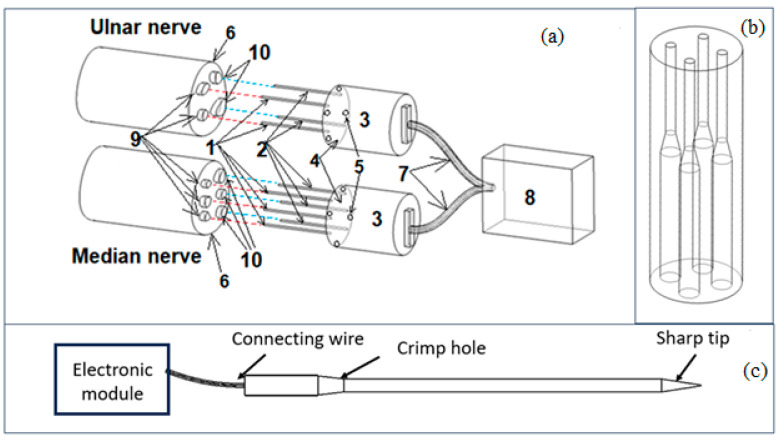
(**a**) Detailed schematic representation of the plug electrodes. (**b**) The support for fixing the needles. (**c**) The components of the needle-type electrodes.

Plug electrodes were designed to have two sets of needle-type electrodes: a set for the acquisition of the signals for commanding the exoprosthesis from the motor fascicles of the stump’s nerves, and another set of needle-type electrodes for stimulating the sensory fascicles of the stump’s nerves with tactile feedback signals from the exoprosthesis to generate tactile sensations to the amputees as presented in Figure 3a.

Plug electrodes are made of biocompatible materials and plated with gold, with the shape shown in Figure 3c, a diameter of 160 microns, and a length of 20 mm. Plug electrodes have been designed so that they can be easily interfaced with a standardized connector for flexible wiring. Plug electrodes contain needle-type electrodes (1), which are implanted in the cross-section of the stump nerves (median nerve and ulnar nerve) in the motor neural fascicles (9), and needle electrodes (2), which are implanted in the cross-section of the stump nerves in the sensory neural fascicles (10). The median nerve contains motor and sensory fascicles corresponding to the thumb, index finger, and middle finger, while the ulnar nerve contains the motor and sensory fascicles corresponding to the ring finger and the little finger.

The needle-type electrodes are fixed in a support (3) made by additive technologies (3D printing) with dimensions adapted to the patient’s nerve from a biocompatible material (gelatin methacryloyl (GelMA)). The support contains a guide tube (4) that is fixed on the nerve and is provided with four holes (5) for suturing (with surgical thread) on the epinerve (6). The needles are connected to the electronic module with conductive wires (7) covered with biocompatible plastic materials (8). Each needle-type electrode has a crimp hole for connecting the electronic module with a conductive wire (Figure 3c).

The support for fixing the needle-type electrodes is shown in Figure 3b and was made by 3D-printing technology from a biocompatible material (gelatin methacryloyl (GelMA)). This support has a diameter adapted to the thickness of the patient’s nerve.

The materials and technology used for the plug electrode fabrication are presented in Table 1. Plug electrodes were made to achieve the selective and bidirectional connection of prostheses with the peripheral nervous system of patients with upper limb amputation (arm/forearm). Plug electrodes were designed to have separate needles for the motor and sensory fascicles of the median and ulnar nerves, so that a distinct needle is inserted into each of the five motor fascicles that control (in a healthy hand) the movements of the five fingers. In the median nerve, there are three motor fascicles that control (in a healthy hand) the thumb, index finger, and middle finger. In the ulnar nerve, there are two motor fascicles that control (in a healthy hand) the movements of the ring finger and the little finger. Thanks to the five needles for acquiring motor signals, plug electrodes offer the patient the advantage of moving each of the five fingers of the prosthesis separately. Compared to plug electrodes, cuff electrodes do not offer this advantage, because they are mounted around the nerve and do not have separate access to each motor fascicle from the median and ulnar nerves. Plug electrodes also offer the patient distinctive tactile sensations from each finger of the prosthesis thanks to the five needles stimulating the sensory fascicles (three from the median nerve and two from the ulnar nerve).

**Figure 4:**
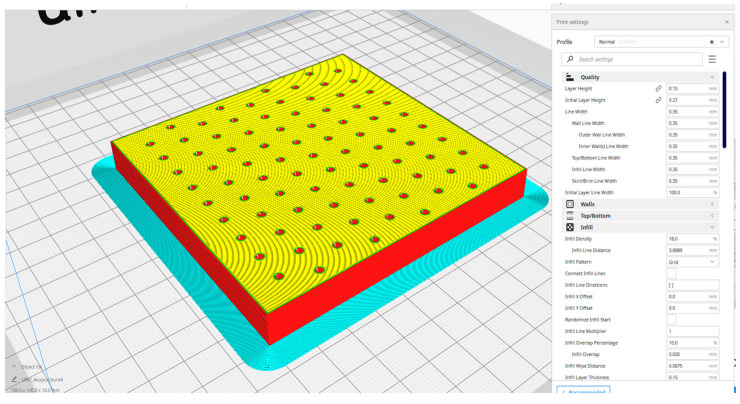
Layout of the needle support used for metal deposition.

The needles used in the experiment (DongBang DB100 Stainless Steel Needles) are made of medical-grade stainless steel, with high tensile strength. The needles are 160 microns thick and 3 cm long. Although they are sterilized, they have undergone a plasma cleaning process before being mounted on a special holder built using the Ultimaker 2+ 3D printer. Figure 4 shows the specially built die to house about 100 needles that will subsequently undergo a sputtering metallization process.

**Figure 5:**
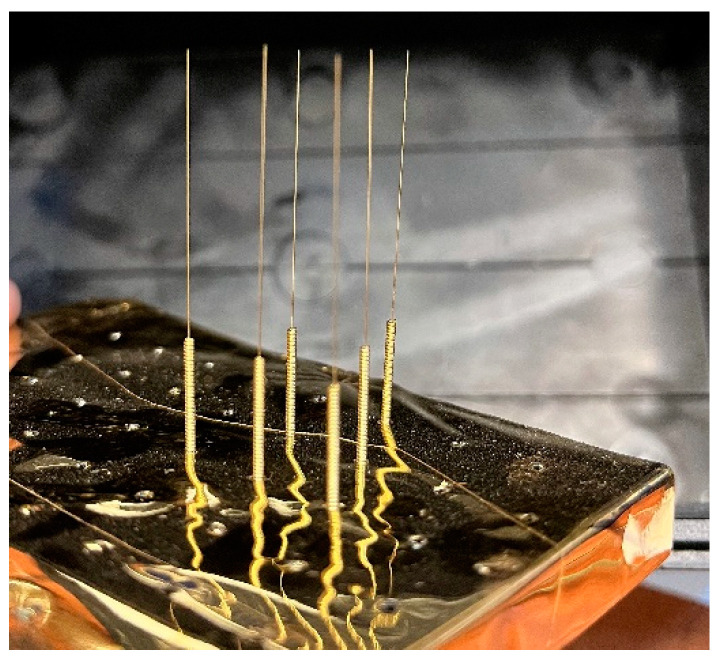
Layout of the needle support used for metal deposition.

**Figure 6:**
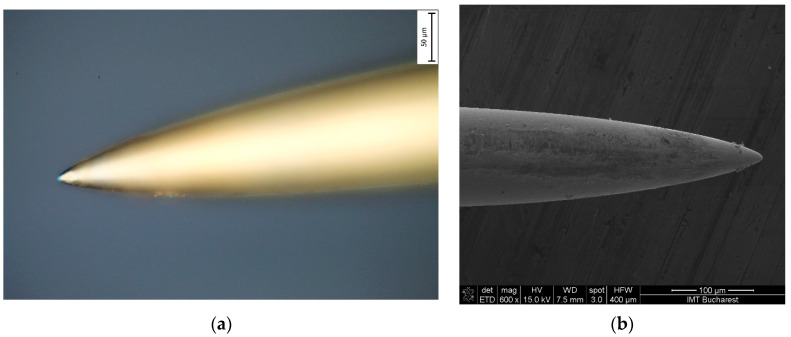
(**a**) Optical image (Leica microscope 50×-BF) of the needle used in our experiments; (**b**) SEM image (Nova™ NanoSEM 630) of the needle used for our plug electrode system.

The resulting ensemble (holder plus needles) is placed in the magnetron sputtering equipment where the first 30 nm thick metallic chromium layer is first deposited, followed by the final 300 nm thick gold layer. Figure 5 shows the assembly after the metallization process. One can observe a very good degree of uniformity. Figure 6a,b show details on the needle tip.

The plug electrodes are implanted together with an electronic module that radio transmits the motor signals acquired from the motor neural fascicles to the exoprosthesis and radio receives from the exoprosthesis the tactile feedback signals that stimulate the sensorial nerve fascicles in which they were implanted.

### 2.2. Electronic Circuits

The electronic circuit used in this experiment was quite similar to the one used in our previous experiments with cuff electrodes presented in [16]. It consists of a front-end circuit necessary to amplify the motor nerve pulse signal which has an amplitude of approximately 5–10 mV, an Atmega328p controller, a Bluetooth communication module, and an inductive power supply module.

Taking into consideration that the conductivity of the myeline cover of the axon could decrease in time, it was decided to use a chain of two amplifiers and establish a control loop on the second amplifier to be able to compensate for this variation. The front-end module consists of a preamplifier realized with an INA118 a low-power, general-purpose instrumentation amplifier with the gain set as G = 100. The second stage of amplification was based on an operational amplifier OPA2340 with small dimensions. The first half of the circuit was used as a voltage follower, and the second half as an amplifier with a gain of G2 = 10. The value of 5 mV considered an acceptable value for the nerve pulse amplitude was thus raised to approximately 5V, a level usable for the input of any controller.

**Figure 7:**
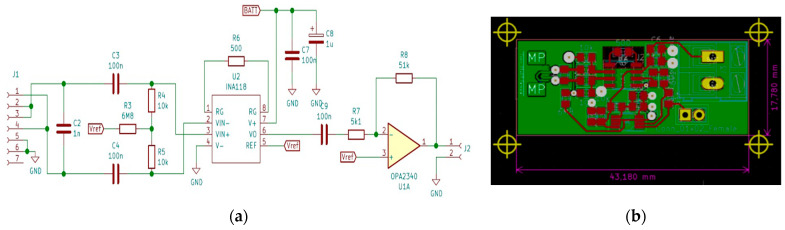
(**a**) The circuit diagram of the front-end module and (**b**) the front-end PCB.

The diagram of the designed front-end circuit and the circuit itself are presented in Figure 7a,b.

To process and wirelessly transmit the acquired and amplified signals an ARDUINO Mini module and an HC-05 Bluetooth shield were used.

The power supply of the implantable circuit was provided by an inductive circuit consisting of two main circuits.

**Figure 8:**
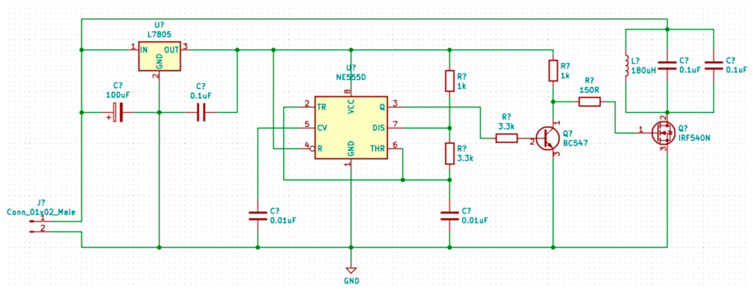
Electrical diagram of generator module.

The generator consists of a voltage stabilizer (IC2 7805), an oscillator (IC1 BM 555), a two-stage amplifier (transistors T1 BC547 and T2 BU549N), and the coil as presented in Figure 8.

**Figure 9:**
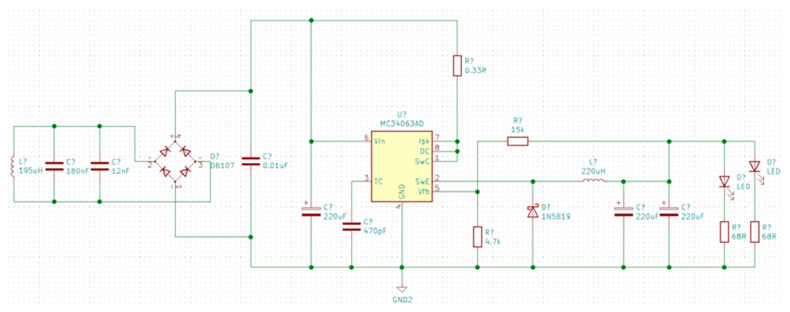
Electrical diagram of receiver module.

The receiver consists of a rectifying bridge BR1 and integrated circuit IC3, MC 34063 which is presented in Figure 9.

**Figure 10:**
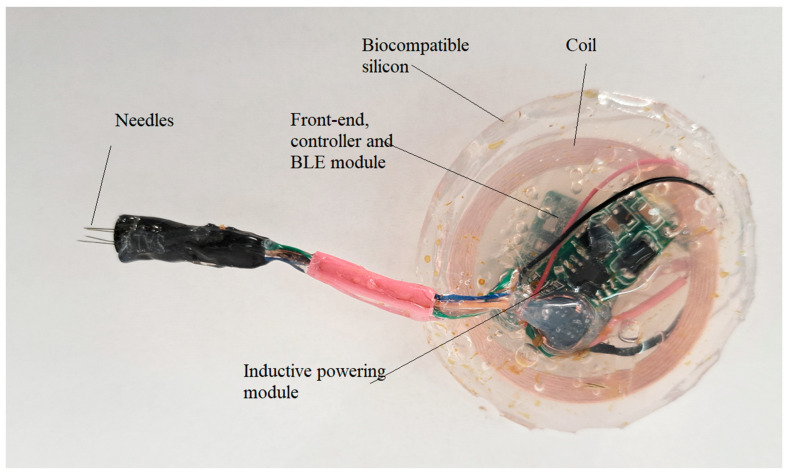
The module equipped with plug electrode designed to harvest signals from the neuronal fascicle.

**Figure 11:**
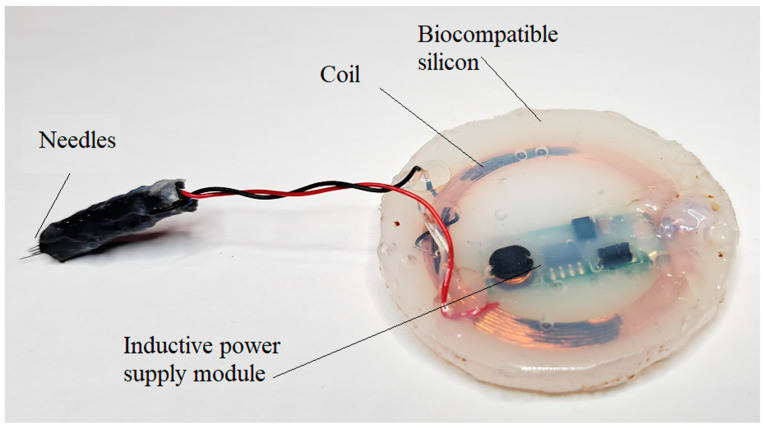
The module equipped with plug electrode designed to send signals to the neuronal fascicle.

For the tests, two modules were prepared. One module to send signals and one module to receive signals. Both modules were encapsulated in biocompatible silicon (DOWSIL ^TM^ 3140 RTV Coating) and are presented in Figure 10 and Figure 11.

## 3. Testing the Plug Electrodes

### 3.1. Laboratory Testing

#### 3.1.1. Electrochemical Characterization In Vitro

The electrical properties of the gold-coated stainless steel needle were determined by measuring impedance between 0.1 Hz–100 kHz and 10 mV amplitude in a saline solution (9 gNaCl/1000 mL water) using a Tacussel-Radiometer Analytical SA France (potentiostat/galvanostat-; PGZ 100), controlled by Volta-Master v.4 software. A three-electrode configuration cell consisting of a gold-coated stainless steel needle as a working electrode, a Ag/AgCl electrode as a reference, and a platinum electrode as a counter was used. The cyclic voltammograms were performed at a 50 mv/s sweep rate during immersion of the gold-coated stainless steel needle in saline solution expressed versus Ag/AgCl. The cyclic voltammetry was started from a negative direction −0.5 V versus Ag/AgCl to a positive direction +0.6 V vs. Ag/AgCl. All experiments were made at a cell temperature of 37.5 °C ± 0.5 °C. In vitro accelerated aging tests of the working electrodes were performed for one month at 37.5 °C ± 0.5 °C with the back end of the stainless steel needle connector outside of the solution. The gold-coated stainless steel needles were immersed in an ultrasound bath for 2, 4, and 15 min. Electrical Impedance was used for measuring before and after ultrasound treatment to investigate any changes in the gold-coated stainless steel. The experiments were repeated after 20 days of immersion of the needles in saline solution at 37.5 °C ± 0.5 °C and each day was completed with the initial volume if the solution was evaporated. The temperature was rigorously controlled throughout the experiment. The tests were carried out on two different needles at the same time to see if the behavior was repeatable.

#### 3.1.2. Testing the Thermal Impact on Tissue of Inductive Charging Device

**Figure 12:**
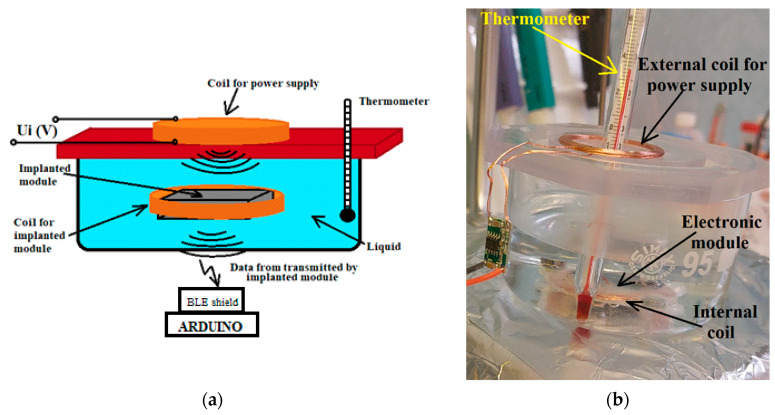
(**a**) The test platform architecture for the electronic module powered by electromagnetic induction; (**b**) monitoring the temperature of the liquid during the functioning of the electronic module powered by electromagnetic induction.

There were serious concerns regarding a possible thermal effect on the patient tissue due to the inductive power supply of the electronic module. To verify if any problems occurred during the in vivo experiments, the special setup presented in Figure 12 was developed and several tests were conducted.

### 3.2. Testing the Plug Electrodes In Vivo

The electronic modules equipped with plug electrodes were tested in one animal experiment in 2023. The studies included two specimens of Sus scrofa domesticus (domestic pig), weighing approximately 50 kg each. The pig experimental model was used for its similarity to humans in nerve diameter, conduction, and impulse transmission.

The experiments had several goals:

- To verify if a specific nerve fascicle could be stimulated without affecting the neighboring fascicles;
- To verify that neuro-signals could be acquired from a specific nerve fascicle without being affected by the signals from neighboring fascicles;
- To verify if needles with a diameter below 0.2 μm are not damaging the nerve;
- To verify if the coating with silicon polymer DOWSIL 3140 is reliable and is not generating rejection phenomena and/or inflammation of tissue;
- To verify if the inductive power supply system is functional when it works with living tissue well.

**Figure 13:**
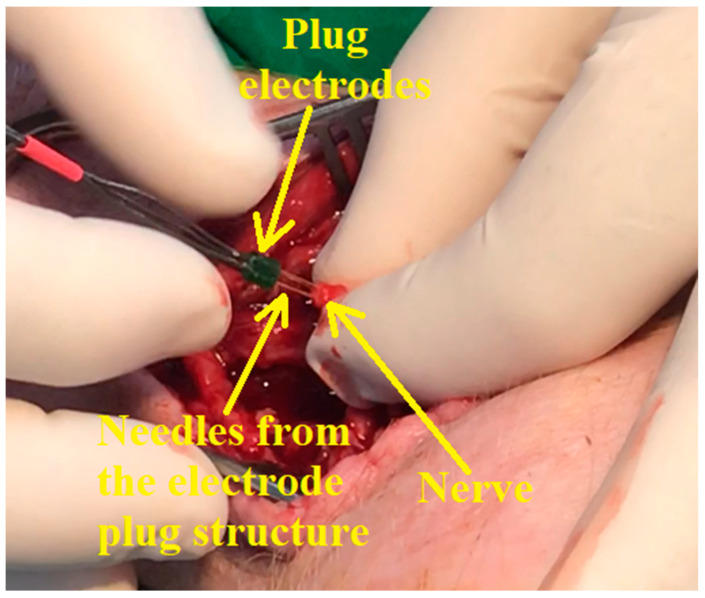
The implantation of the plug electrodes into the pig’s nerve.

To reduce stress, the pigs were housed in the experimental research facility and monitored for 48 h before the interventions. Free access to water was allowed, while feeding was stopped 18 h before anesthesia. The methodology in the two animal experiments adhered to the guidelines outlined in the Public Health Service Policy on the Humane Care and Use of Laboratory Animals (2015). All surgical procedures were performed under general anesthesia, as follows: rapid administration in the cervical area of the trapezius muscle of intramuscular premedication using Ketamine 20 mg/kg and Xylazine 1 mg/kg for deep sedation and immobilization. After 10 min, venous access was performed using an 18-gauge catheter in the left lateral auricular vein. For induction of anesthesia, Propofol 3 mg/kg was administered intravenously, followed by intubation using a long-bladed laryngoscope, stylet, and 5.5 tube. After obtaining an appropriate level of anesthesia, the experimental animal is positioned in such a way as to facilitate the approach to the deep peroneal nerve. One of the pictures taken during the surgery is presented in Figure 13.

All the procedures from the animal experiments were approved by the Faculty of Veterinary Medicine Ethics Committee and by the Romanian Sanitary Veterinary Directorate and adhered to the guidelines outlined in Directive 2003/65/EC of the European Parliament and of the Council of 22 July 2003 amending Council Directive 86/609/EEC on the approximation of laws, regulations and administrative provisions of the member states regarding the protection of animals used for experimental and other scientific purposes [39].

The module for harvesting the neural signals was implanted on the first subject and the module for stimulation on the second subject.

## 4. Results Obtained

### 4.1. Results for Electrochemical Characterization In Vitro

Although implantable materials are dominated by indium tin oxide (ITO) because of its high biocompatibility, when considering other factors, such as resistivity and ease of fabrication, gold seems to be the material of choice for electrode sites on long-term implantable microelectrodes because of its low resistivity, ease of fabrication and availability. For implantable metal materials, corrosion can be a problem as these materials in contact with the blood can over time release remnants of metal ions that can cause allergic reactions or other problems related to biocompatibility. The existing toxicity studies on these materials (gold, platinum, iridium, titanium) for in vivo implantation have shown acceptable cell viability, with values of over 75% [40,41].

Furthermore, it is well known that stainless steel possesses stability against corrosion and the capability to self-heal in the event of mechanical or chemical damage. However, the level of corrosion resistance greatly relies on the specific type of stainless steel utilized [34]. A comprehensive examination was conducted to investigate the stability of AISI 316 L stainless steel regarding pitting corrosion by utilizing its transfer function. The study explored the irregularity of the localized current distributions of corrosion in 316 L stainless steel when exposed to a -NaCl solution [42]. To assess the adhesion of the gold coating on the stainless steel substrate, an ultrasound procedure was conducted after various immersion times, specifically on the first day and 21st day of immersion. This procedure allowed for evaluating the stability of the coating over time. Following ultrasound treatment for 2, 4, and 15 min on the first day and 21st day of immersion, the gold-coated needles were examined by recording CVs (cyclic voltammetry) and electrochemical impedance spectra (EIS) in a saline solution at a temperature close to the human body. EIS and CV were measured before and after treatment to investigate changes in the coatings.

**Figure 14:**
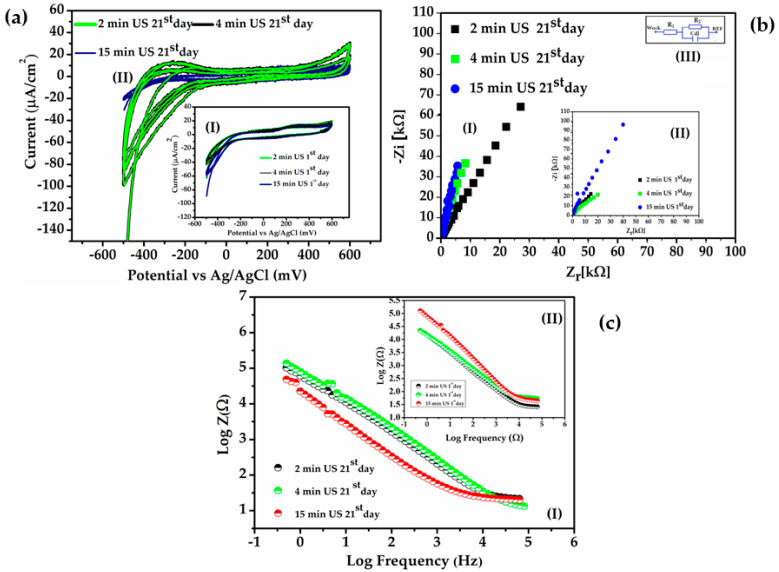
(**a**) CVs (-600 mV+600 mV) in the saline solution (9 g/L) of the gold-coated needles stainless steel electrode after different times of ultrasonication: first day (curves (**I**)) and after 21st day of the experiments (curves (**II**)); (**b**) Nyquist diagrams (-Zi vs. Zr) of the gold-coated needles stainless steel electrode after different time of ultrasonication: 21st day (curves (**I**)) and first day of the experiments (curves (**II**)); inset (**III**): the equivalent circuit: resistance charge transfer (R_2_), capacitive dielectric double layer (C_dl_), and resistance solution (R1); (**c**) EIS (Bode plots) of gold-coated needles stainless steel electrodes on (**I**) 21 st day of immersion in saline solution and US times (**II**), after first day and US times (**II**) as a function of frequency decimal logarithm.

The CVs after 2 and 4 min showed no apparent changes, even after 15 min of ultrasound treatment at high power (Figure 14a, curves I). The stability of the coating needles was also assessed after their immersion in saline solution for 21 days at a temperature of 37.5 °C. Subsequently, the needles were subjected to ultrasound treatment again for 2, 4, and 15 min. The CVs did not exhibit significant changes (Figure 14a, curves II), indicating the stability of the gold-coated needles at human body temperature if they remain implanted for 21 days. The fitting of the experimental measurements for Nyquist plots (Zi vs. Zr) data with the electrical circuit model R_1_R_2_C, presented in Figure 14b, consists of the parameters Rı (solution resistance), R_2_ (charge transfer resistance at the gold-coated stainless steel electrode), and C dl (double-layer capacity).

In Figure 14b(I,II), the absence of the semicircle in the Nyquist diagrams (Zi vs. Zr) indicates that no salts have been deposited on the surface of the gold, and the solution resistance is mainly dominated by that of the saline solution (R1). However, a slight decrease in conductivity was observed after ultrasonication and prolonged immersion in saline solution, with no significant modifications.

Figure 14c shows the EIS spectra (Log Z versus Log frequency) of the needle electrode over a span of 21 days of experiments after ultrasonication treatments. Observing Figure 14b, the impedance exhibits a minor increase after 21 days. The slope of impedance in Figure 14c shows negligible modification over time at cut-off frequencies (the intersection between the capacitive part and the resistive part).

Therefore, the gold-coated needles demonstrate consistency over time, maintaining a constant slope of impedance spectra at 1 kHz, even after ultrasound treatment. After 21 days of electrode immersion in a solution with 37.5 °C and 15 min of ultrasound treatment, a slight increase in surface roughness was observed, accompanied by a decrease in resistance at electronic transfer to the electrode (R_2_), which is likely due to water adsorption. However, these changes were not very pronounced for the gold-coated needle electrodes during the immersion period, as also observed in other studies that investigated the long-term stability of gold in saline or phosphate solutions [43,44]. Determining the frequency of 1 kHz in vitro tests for implantable electrodes is important for evaluating their electrochemical behavior and tissue interaction, ensuring measurement stability, and achieving comparability of data across different studies and research [45,46]. The impedance at 1 kHz (Figure 14c(I,II)) remained unchanged after 21 days of immersion of gold-coated needles in saline solution at body temperature and after ultrasound treatments. The slight differences in R_1_ and the fluctuation in impedance at low frequencies could be caused by experimental errors or changes in the surrounding environments. This indicates that the gold-coated needles are electrochemically stable for a period of 21 days, even when exposed to bodily fluids.

### 4.2. Results of In Vivo Testing

The great challenge in conducting the measurement was to provide electrical power to the module. Fixing the coil in the right position on the pig when is awake it was a great challenge. To overcome this inconvenience the two subjects were sedated while the measurements were conducted. During sedation the animal is generally unaware of its surroundings but, contrary to unconsciousness, can be stimulated by different stimuli [47].

The functionality of the inductive power supply module was verified on the first day, the fifth day, and the tenth day after the surgery, before the modules were extracted. The verification consisted of fixing the coil on the sedated pig and verifying if the Bluetooth module was connecting, maintaining the connection with the smartphone and signals were received in a continuous mode for the first module, and if the pig was moving the leg when the coil is placed in the right position for the second module. During all the stages of verifications, we were able to connect the smartphone with the implanted module and to receive signals from the first module as well as to induce movement of the pig leg with the second module. These results prove that for both implanted modules the inductive power system functioned well.

There were previous experiments in regard to the wireless reception/transmission of neural signals, using cuff electrodes as presented in the articles [16,46,48,49]. In these experiments, it was observed that a stimulus applied on the nerve was received by different fascicles and generated a complex movement of both pig toes and pig leg [Video S1]. A similar phenomenon was observed on the reception path, the recorded signals being noisy due to the pulses harvested from different fascicles of nerves. At this step of experiments, we focused on the capability of the plug electrodes to receive and transmit selectively signal from/to the fascicle of nerves.

To demonstrate that it is possible to target a specific fascicle of nerve, the doctors selected and implanted the needles in the specific fascicle which was responsible for pig leg movement. During all three stages of testing (first, fifth, and tenth day), it was observed that the pig leg was moving but the toes were not.

**Figure 15:**
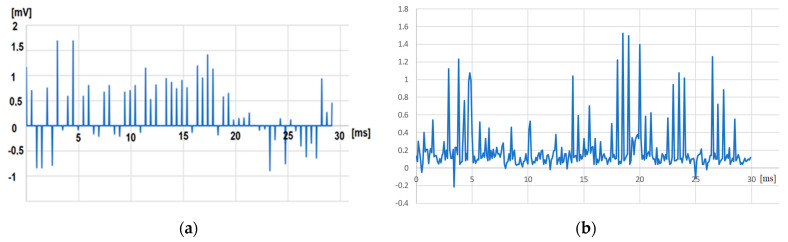
(**a**) Signals acquired with implanted module equipped with plug electrodes. (**b**) Signals acquired with implanted module equipped with cuff electrodes.

In regard to proving the selective recording of signals, the testing was much more challenging since during the recording, the pig was sedated and it was not possible to associate a pulse with a specific trigger; however, during the sedation, the brain was continuously sending random pulses, with lower amplitude [Video S2]. The only way to assume that the implanted module received signals only from the specific fascicle selected by the doctors was to compare the signals recorded with the cuff electrode presented in Figure 15a with the signals recorded with the plug electrodes as presented in Figure 15b. During all three steps of the experiments, the first implanted module equipped with plug electrodes received a less noisy signal in comparison with the signals received by the module equipped with cuff electrodes [16]. Although this is not direct evidence of the selective recording, it could be considered an indicator that the module is recording signals only from the fascicles where the needles were inserted.

The fact that after 10 days the implantable modules were still functional and capable of transmitting/recording impulses to/from the nerve was a clear indicator that the needles with a diameter below 200 μm were not damaging the nerves; however, after the implanted modules were extracted, the nerve and the tissue around the implanted module were thoroughly inspected and the doctors did not find any inflammations, infections or modifications which could have led to module rejection. In addition, samples of nerves and tissues were sent to the histopathologic analysis to investigate if any modifications that could not be seen during the visual inspections occurred. The reports received from the doctors proved that the nerve was not damaged and there were no signs of tissue modifications.

The modules extracted were analyzed and it was observed that the coating realized with silicon polymer DOWSIL 3140 was not affected by the pig body fluids and their encapsulation was stable and reliable as could be observed in Figure 10 and Figure 11.

5. Conclusions and Further Experiments

The laboratory biocompatibility tests were conducted for only 21 days and, although the results were encouraging, there is an acknowledged need for a continuation of these tests for a longer period to prepare the next step of experiments on human subjects.

The results obtained during the in vivo tests are proving that after a period of 10 days:

- The inductive charging system is working well, providing the required power for the implanted modules;
- The coating with silicon polymer DOWSIL 3140 was not affected by the pig body fluids and did not generate adverse reactions from the pig tissue;
- The needles with a diameter of less than 200 μm do not affect the nerve fascicle;
- The signals transmitted to a specific fascicle of nerves by the plug electrodes do not affect the neighboring fascicles.

There is still significant work to be carried out to prove without any doubt the aspect of selective recording the nerve pulses. Inferential, we may assume that the less nosy signal acquired by the module equipped with a plug electrode is an indicator of the fact that there were recorded pulses only from the fascicles targeted by the doctors; however, this should be thoroughly investigated in the next experiments.

The next stage of the project will involve carrying out in vivo experiments on sheep. This decision was assumed due to several significant advantages:

- The behavior of sheep is less aggressive than the behavior of pigs; consequently, it will be possible to conduct measurements without sedating them, and thus we will be able to have a better understanding of how selective are the plug electrodes;
- Sheepskin is closer in thickness to human skin, compared to pig skin, which is thicker and significantly reduces the amplitude of the signals received from the implantable module. In this regard, we are looking forward to attaching the external inductive powering module to the sheep leg and recording from a safe distance the neural signals.

In the experimental animal of sheep, we maintained the electrodes and electronic modules implanted for a longer period of time (60 days) to investigate if the implanted electrodes are affected by the body fluids, as well as if the nerves where the electrodes were implanted and the tissues around them are affected.

The biocompatibility, toxicity, and pathology tests which will be conducted at the end of the sheep experiment are going to indicate what improvements we need to make and whether we will be able to proceed to the last stage of the project, the implantation of the electrodes on a human patient.

Plug electrodes are the object of the patent with no. A/00837/27.12.2022 and International Extension no. 32350/16.11.2023.

## Figures and Tables

**Table 1:** Plug-type electrode components and parameters.

Type	Component	Dimensions	Technology/Material
Plug electrodes for median and ulnar nerve	Needles	length = 10 mm diameter = 130 µm (tip)—100 µm (upper side)	Stainless steel
	Support for fixing the needles	4–5 mm diameter	
	Guide tube	5 mm diameter, 4 mm length	3D-printed biocompatible polyester/polyetilene
	Needle connector	3 mm	MOLEX 52745-0897
	Connecting wire	20–30 mm	Cable with 7 wires insight

## Data Availability

The data presented in this study are available on request from the corresponding author. The data are not publicly available due to the peculiarities of the software used to record them.

## Supplementary Materials

The following supporting information can be downloaded at: https://www.mdpi.com/article/10.3390/bios14010031/s1. Video S1: stimulation with wireless powered module and Video S2: sigrec 2.
